# Cytoreductive treatment patterns among US veterans with polycythemia vera

**DOI:** 10.1186/s12885-018-4422-6

**Published:** 2018-05-04

**Authors:** Shreekant Parasuraman, Jingbo Yu, Dilan Paranagama, Sulena Shrestha, Li Wang, Onur Baser, Robyn Scherber

**Affiliations:** 1 Incyte Corporation, 1801 Augustine Cut-Off, Wilmington, DE 19803 USA; 2 STATinMED Research, Plano, TX USA; 3 Center for Innovation & Outcomes Research (CIOR), Columbia University, New York, NY USA; 4 Oregon Health and Sciences University, Portland, OR USA; 5 Department of Hematology and Oncology, Mayo Clinic, Scottsdale, AZ USA

**Keywords:** Polycythemia vera, Thrombosis, United States Department of Veterans Affairs, Veterans Health Administration

## Abstract

**Background:**

Polycythemia vera (PV) is a myeloproliferative neoplasm associated with increased thrombotic and cardiovascular risk, which are key contributors to patient morbidity and mortality. The Veterans Health Administration (VHA) is the largest integrative health network in the United States. Available data concerning patients with PV in this population are limited.

**Methods:**

This retrospective observational study evaluated the characteristics, management, and outcomes of patients with PV in the VHA Medical SAS® Dataset (October 1, 2005, to September 30, 2012). Inclusion criteria were ≥ 2 claims for PV (ie, PV diagnostic code was recorded) ≥30 days apart during the identification period, age ≥ 18 years, and continuous health plan enrollment from ≥12 months before the index date until the end of follow-up. All data were analyzed using descriptive statistics.

**Results:**

The analysis included 7718 patients (median age, 64 years; male, 98%; white, 64%). The most common comorbidities before the index date were hypertension (72%), dyslipidemia (54%), and diabetes (24%); 33% had a history of smoking. During the follow-up period (median, 4.8 years), most patients did not receive treatment with cytoreductive therapy, including phlebotomy (53%), or antiplatelet agents, such as aspirin (57%). The thrombotic and cardiovascular event rates per 1000 patient-years were 60.5 and 83.8, respectively. Among patients who received cytoreductive treatment, the thrombotic event rate was 48.9 per 1000 patient-years. The overall mortality rate was 51.2 per 1000 patient-years.

**Conclusion:**

The notable rates of thrombotic and cardiovascular events observed in this analysis, even among patients receiving cytoreductive treatment, highlight the important unmet clinical needs of patients with PV in the VHA.

**Electronic supplementary material:**

The online version of this article (10.1186/s12885-018-4422-6) contains supplementary material, which is available to authorized users.

## Background

Polycythemia vera (PV) is a myeloproliferative neoplasm (MPN) [1] that affects > 140,000 patients in the United States [2]. In the National Cancer Institute−sponsored Surveillance, Epidemiology, and End Results cancer registry, the incidence of PV per 100,000 persons increased with advancing age (0.1 for < 34 years, 0.7 for 35–49 years, 2.4 for 50–74 years, and 5.3 for > 75 years) and was higher for men (1.3 vs 0.8 for women) and whites (1.1 vs 0.7 for African Americans and 0.8 for other race groups) [3]. Analyses of PV patient populations have estimated that arterial and venous thrombotic events occur at rates of 7 to 21 and 5 to 20 per 1000 person-years, respectively [4–7]. Thrombotic and cardiovascular events are among the leading causes of death in patients with PV [8], contributing to lower overall survival compared with age- and sex-matched members of the general population [9].

The treatment goals for patients with PV focus primarily on preventing or managing thrombotic and bleeding complications [10]. The European Collaboration on Low-Dose Aspirin in Polycythemia Vera (ECLAP) study demonstrated that treatment with low-dose aspirin was associated with reduced risk of thrombotic events and death from cardiovascular causes [11]. Results from the Cytoreductive Therapy in Polycythemia Vera (CYTO-PV) trial supported treatment with phlebotomy to maintain hematocrit levels < 45% and reduce the risk of cardiovascular events and deaths resulting from thrombotic or cardiovascular events [12]. In addition, some patients benefit from cytoreductive treatment with hydroxyurea [13], interferon-α [14], or ruxolitinib [15, 16]. Ruxolitinib was approved by the US Food and Drug Administration (FDA) in 2014 for patients with PV who have had an inadequate response to or are intolerant of hydroxyurea, and it remains the only pharmaceutical agent approved by the FDA for the PV setting [17].

Real-world data on patient characteristics and clinical management of patients with PV help inform the understanding of the population and identification of unmet clinical needs. The Veterans Health Administration (VHA), the largest integrated health-care system in the United States, maintains patient records for US veterans receiving care in Veterans Integrated Service Networks. This is the first study to describe the demographics, clinical characteristics, management, and thrombotic and cardiovascular event rates of patients with PV in the VHA population.

## Methods

### Study design

**Fig. 1:**
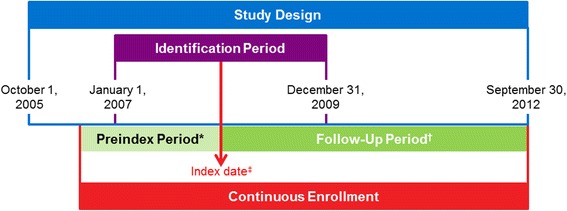
Study design. PV, polycythemia vera. * ≥ 12 months. ^†^Patients were followed up from the index date until the date of the earliest event: death, disenrollment, or end of the study period. ^‡^The index date was defined as the date of each respective patient’s first claim with a PV diagnosis during the identification period

This retrospective, observational study analyzed longitudinal data from the VHA Medical SAS® Dataset catalogued between October 1, 2005, and September 30, 2012 **(**Fig. 1**)**. The VHA data set included deidentified patient-level data from 21 Veterans Integrated Service Networks linking inpatient, outpatient, pharmacy, laboratory, enrollment, and vital sign databases.

### Patients

Patients who had ≥2 claims (ie, diagnostic code was recorded) for PV (defined as International Classification of Diseases, Ninth Edition, Clinical Modification [ICD-9-CM] code 238.4) ≥30 days apart during the identification period (January 1, 2007, to December 31, 2009) were included in the analysis. Additional inclusion criteria were age ≥ 18 years old on the index date (defined as the date of each patient’s first claim with a PV diagnosis during the identification period) and continuous health plan enrollment with medical and pharmacy benefits from ≥12 months before the index date (ie, pre-index period) until the end of follow-up. Patients were followed from the index date until the date of death, date of disenrollment, or end of the study period (September 30, 2012), whichever occurred first.

### Assessments and analyses

Patient demographics at index were reported. Assessments of pre-index patient characteristics included comorbidities, mean Deyo-Charlson Comorbidity Index (a weighted score [range, 1–6] of disease severity calculated using 19 conditions) [18], mean Chronic Disease Score (an aggregate comorbidity measure based on medication use) [19], and patient histories of thrombotic and cardiovascular events.

Patient records were reviewed for PV treatments and concomitant medications received; hematocrit level and white blood cell (WBC) count measures; and thrombotic, cardiovascular, and mortality events that occurred between the index date and the end of the follow-up period. Among patients with evaluable laboratory data, the proportions of patients with elevated hematocrit and WBC counts were recorded at the pre-index period and annually during the follow-up period. Hematocrit levels ≥45% and WBC counts ≥11 × 10^9^/L were considered elevated. Thrombotic and cardiovascular events were not mutually exclusive (acute myocardial infarction, ischemic stroke, and transient ischemic attack spanned both categories), and were identified by ICD-9-CM diagnosis codes recorded in patient claims (Additional file 1: Table S1).

All data were analyzed using descriptive statistics.

## Results

### Baseline patient demographic and clinical characteristics

In all, 7718 patients with PV were included in the analysis. Most patients were ≥ 60 years of age (70.7%), male (97.9%), and white (63.9%; Table 1). Before the index date, hypertension (71.7%), dyslipidemia (54.2%), and diabetes (24.0%) were the most common comorbidities. The mean (SD) Chronic Disease Score was 6.3 (4.2). A notable proportion of patients experienced thrombotic (arterial, 8.8%; venous, 4.5%) or cardiovascular (17.0%) events within a year before the index date. Among patients with available laboratory data, ≥1 elevated hematocrit level or WBC count was reported in 90.2% and 36.5% of patients, respectively, within a year before the index date. One third (33.1%) of patients had a history of smoking.

**Table 1:** Patient Demographics and Clinical Characteristics

	Patients With PV (*N* = 7718)
Age at index date,^a^ y	
Mean (SD)	65.9 (11.3)
Median (range)	64 (21–93)
Age, n (%)	
18–45 y	256 (3.3)
46–59 y	2004 (26.0)
≥ 60 y	5458 (70.7)
Sex, n (%)	
Male	7557 (97.9)
Race/ethnicity, n (%)	
White	4932 (63.9)
Black	404 (5.2)
Hispanic	275 (3.6)
Other^b^	134 (1.7)
Unavailable^c^	1973 (25.6)
Comorbid conditions, pre-index^a^	
Deyo-Charlson Comorbidity Index, mean (SD)	1.4 (1.7)
Chronic Disease Score, mean (SD)	6.3 (4.2)
Most frequent comorbidities, n (%)	
Hypertension	5531 (71.7)
Dyslipidemia	4181 (54.2)
Diabetes	1851 (24.0)
Bleeding^d^	669 (8.7)
Major	512 (6.6)
Minor	262 (3.4)
History of smoking,^e^ n (%)	2551 (33.1)
Elevated, pre-index,^a^ n/N^f^ (%)	
Hematocrit level	5608/6214 (90.2)
WBC count	2365/6477 (36.5)
Thrombotic events,^d^ pre-index,^a^ n (%)	
Arterial thrombosis	681 (8.8)
Venous thrombosis	349 (4.5)
Cardiovascular events pre-index,^a^ n (%)	1315 (17.0)

*ICD-9-CM* International Classification of Diseases, Ninth Edition, Clinical Modification, *PV* polycythemia vera, *WBC* white blood cell

^a^The index date was defined as the date of each respective patient’s first claim with a PV diagnosis during the identification period

^b^Hawaiian or other Pacific Islander (0.8%), Alaska native or American Indian (0.6%), Asian (0.4%)

^c^Missing (21.1%), declined to answer (3.1%), or unknown by patient (1.4%)

^d^Number of patients with ≥1 event. Patients may be counted in > 1 subcategory

^e^Smoking status was determined through *ICD-9-CM* code 305.1, V15.82

^f^Elevated defined as hematocrit level ≥ 45%, WBC count ≥11 × 10^9^/L; N represents patients with evaluable laboratory data

### Laboratory values during follow-up

The median duration of follow-up was 4.8 years. Among patients with available laboratory data, ≥1 elevated hematocrit level was reported in 85.9% of patients during the first year of follow-up and in 91.6% by the end of follow-up **(**Table 2**)**. The proportion of patients who experienced ≥1 elevated WBC count was 34.4% during the first year of follow-up and 51.4% by the end of follow-up. Many patients experienced ≥2 elevated hematocrit levels (86.7%) or WBC counts (37.3%) during follow-up.

**Table 2:** Patients With Elevated Hematocrit Levels and WBC Counts During the Follow-Up Period

Laboratory Value, n/N (%)^a^	Patients With PV
≥1 elevated hematocrit level^b^	
12-month follow-up period	5350/6230 (85.9)
24-month follow-up period	6167/6974 (88.4)
36-month follow-up period	6397/7115 (89.9)
48-month follow-up period	6492/7140 (90.9)
Entire follow-up period	6544/7142 (91.6)
≥1 elevated WBC count^c^	
12-month follow-up period	2163/6280 (34.4)
24-month follow-up period	2851/6966 (40.9)
36-month follow-up period	3239/7113 (45.5)
48-month follow-up period	3494/7136 (49.0)
Entire follow-up period	3671/7137 (51.4)

*PV* polycythemia vera, *WBC* white blood cell

^a^N represents patients with evaluable laboratory data

^b^Defined as hematocrit ≥45%

^c^Defined as WBC count ≥11 × 10^9^/L

### Cytoreductive therapy use during follow-up

During follow-up, only 23.2% of patients had documentation of any pharmacologic cytoreductive treatment **(**Table 3**)**. Among those who received cytoreductive therapy (*n* = 1787), hydroxyurea was used most often (86.7% [1550/1787]). Only 32.8% of all patients had a record of treatment with phlebotomy, and more than half of all patients (53.0%) had no record of cytoreductive treatment or phlebotomy. Overall, fewer than 1 in 10 patients (8.9%) received both cytoreductive treatment and phlebotomy. The most common concomitant medications were antihypertensive agents and antilipid/anticholesterol agents, each received by a majority of patients; anticoagulants were received by 17.0% of patients **(**Table 3**)**.

**Table 3:** PV Treatment Patterns and Concomitant Medications During the Follow-Up Period

Treatment, n (%)	Patients With PV (*N* = 7718)
PV-related treatment^a^	
Pharmacologic cytoreductive treatment^b^	1787 (23.2)
Hydroxyurea	1550 (20.1)
Anagrelide	206 (2.7)
Radiophosphorus	132 (1.7)
Interferon or PEGylated interferon	90 (1.2)
Busulfan	15 (0.2)
Phlebotomy^c^	2531 (32.8)
Pharmacologic cytoreductive treatment and phlebotomy	688 (8.9)
No cytoreductive treatment or phlebotomy	4088 (53.0)
Aspirin^d^	1815 (23.5)
Antiplatelet agents, not including aspirin	1511 (19.6)
Common concomitant medications	
Antihypertensive agents	6671 (86.4)
ACE inhibitors	4226 (54.8)
Antilipid/anticholesterol agents	4882 (63.3)
Antidiabetic agents	2163 (28.0)
Anticoagulants	1313 (17.0)
Inotropic agents	580 (7.5)
Antiarrhythmic agents	362 (4.7)

*ACE* angiotensin-converting enzyme, *PV* polycythemia vera

^a^Ruxolitinib received approval from the US Food and Drug Administration for patients with PV who have had an inadequate response to or are intolerant of hydroxyurea after the end of the study period

^b^Patients receiving pharmacologic cytoreductive treatment may have also received phlebotomy

^c^Patients receiving phlebotomy may have also received pharmacologic cytoreductive treatment

^d^Includes both prescribed and over-the-counter aspirin and may underrepresent actual aspirin use

### Thrombotic and cardiovascular events during follow-up

Thrombotic and cardiovascular events occurred in 22.9% and 30.1% of patients, respectively **(**Table 4**)**. The rate of thrombotic events during follow-up was 60.5 per 1000 patient-years. Arterial thrombotic events were approximately twice as common as venous events; the most common arterial and venous thrombotic events were ischemic stroke (10.7%) and deep vein thrombosis (6.9%), respectively **(**Table 4**)**. The rate of thrombotic events was 48.9 per 1000 patient-years among patients who received cytoreductive treatment before the event. Cardiovascular events occurred at a rate of 83.8 per 1000 patient-years. On average, the first thrombotic and cardiovascular events occurred 1.4 years after the index date. Overall, 1776 (23.0%) patients died during follow-up, which was a mean 2.8 years after the index date. The mortality rate was 51.2 per 1000 patient-years **(**Table 4**)**.

**Table 4:** Thrombotic Events, Cardiovascular Events, and Deaths Occurring During the Follow-Up Period

Event	Patients With ≥1 Event, n (%)	Mean Time to First Event, y	Patient-Years^a^	Event Rate, per 1000 Patient-Years^b^	95% CI of Event Rate
Among all patients with PV (N = 7718)					
Thrombotic event	1771 (22.9)	1.4	29,276.2	60.5	57.7–63.4
Arterial	1275 (16.5)	1.5	30,874.3	41.3	39.1–43.6
Ischemic stroke	825 (10.7)	1.4	32,216.2	25.6	23.9–27.4
Transient ischemic attack	344 (4.5)	1.6	33,609.5	10.2	9.2–11.4
Acute myocardial infarction	224 (2.9)	2.3	34,167.1	6.6	5.8–7.5
Peripheral arterial thrombosis	109 (1.4)	2.0	34,364.7	3.2	2.6–3.8
Venous	678 (8.8)	1.4	32,621.4	20.8	19.3–22.4
Deep vein thrombosis	535 (6.9)	1.4	32,992.0	16.2	14.9–17.7
Pulmonary embolism	234 (3.0)	1.7	34,029.2	6.9	6.1–7.8
Superficial thrombophlebitis	42 (0.5)	1.9	34,529.8	1.2	0.9–1.7
Cardiovascular event	2325 (30.1)	1.4	27,750.5	83.8	80.4–87.3
Heart failure	1442 (18.7)	1.5	30,730.4	46.9	44.6–49.4
Ischemic stroke	825 (10.7)	1.4	32,216.2	25.6	23.9–27.4
Transient ischemic attack	344 (4.5)	1.6	33,609.5	10.2	9.2–11.4
Acute myocardial infarction	224 (2.9)	2.3	34,167.1	6.6	5.8–7.5
Unstable angina	185 (2.4)	2.3	34,193.5	5.4	4.7–6.3
Percutaneous coronary intervention	78 (1.0)	2.0	34,444.8	2.3	1.8–2.8
Coronary artery bypass graft	7 (0.1)	2.0	34,624.2	0.2	0.1–0.4
Mortality	1776 (23.0)	2.8	34,684.6	51.2	48.9–53.6
Among patients with PV receiving cytoreductive treatment (*n* = 1522)					
Thrombotic event	295 (19.4)	2.2	6032.3	48.9	43.6–54.8
Among patients with PV not receiving cytoreductive treatment (*n* = 6196)					
Thrombotic event	1476 (23.8)	1.2	23,243.8	63.5	60.3–66.8

*PV* polycythemia vera

^a^Up to the first respective event after diagnosis or end of the study period, whichever occurred first

^b^Rate per 1000 patient-years was defined as 1000 × number of patients with first event/patient-years of total patients with PV

## Discussion

In this analysis of 7718 patients diagnosed with PV in the VHA population, there was a substantial burden of thrombotic and cardiovascular events. During follow-up, nearly a quarter of patients had a thrombotic event, and almost one-third experienced a cardiovascular event. The thrombotic event rate (60.5 per 1000 patient-years) was higher than rates reported for cohorts of patients with PV in the general population (14.3 to 38 per 1000 patient-years) [5–7], even among high-risk patients (diagnosis before 2005, 40.1 per 1000 patient-years; after 2005, 29.3 per 1000 patient-years) [20].

There are several plausible contributors to the elevated thrombotic event rate in the VHA population. First, the VHA population may inherently have more risk factors for thrombotic events. Patients had a notable comorbid disease burden at baseline, and the prevalence of traditional cardiovascular risk factors such as hypertension, dyslipidemia, diabetes, and smoking [21] was high. Although not measured in our analysis, the prevalence of psychological comorbidities (eg, adjustment disorder, anxiety, depression, posttraumatic stress disorder, substance use disorder) [22] and other psychosocial issues (eg, homelessness) [23] are also elevated in the VHA patient population, which may complicate management of thrombotic and cardiovascular events. Finally, the catchment area overseen by VHA providers may be larger than the area covered by some traditional hematology providers, which could confound travel logistics and scheduling for some patients, thereby impeding some standard practices (eg, frequent phlebotomy, coordination of care, obtaining outside laboratory tests) [24].

Although the care provided during the follow-up period may have been appropriate by the standards of the time, current standard practice based on more recent evidence may be associated with improved patient care. Of interest, 3 in 4 patients had no documentation of any pharmacologic cytoreductive treatment, and more than half had no documentation of pharmacologic cytoreductive treatment or phlebotomy. The low cytoreductive treatment rate in this population may explain why 9 in 10 patients had elevated hematocrit levels and 1 in 3 had elevated WBC counts. These findings are important; the CYTO-PV study indicated that elevated hematocrit and WBC counts were associated with increased risk of PV-related clinical complications [12, 25]. The cytoreductive treatment patterns observed in our study may be related to the available evidence at the time. For example, CYTO-PV study results demonstrating the clinical benefit of hematocrit maintenance < 45% [12] and WBC counts < 11 × 10^9^/L [25] were not published until after the study period of this analysis. However, some data concerning the clinical benefits of cytoreductive therapy, in particular hydroxyurea, predated the study period [13, 26]. It is interesting that few patients had documented treatment with aspirin or other antiplatelet therapy. This may be a consequence of anticoagulant use or an artifact of low-dose aspirin being available over the counter (ie, may not be reflected in the medication dispensing forms). Findings from the ECLAP study published in 2004 demonstrated that low-dose aspirin reduced the risk of cardiovascular events in patients with PV [11]. Recent data suggest that patients with PV and hypertension who are treated with angiotensin-converting enzyme (ACE) inhibitors may require less cytoreductive treatment to control hematocrit levels compared with those who are treated with other antihypertensive agents [27]. The current analysis found that the majority of patients with PV received an ACE inhibitor during follow-up, but usage rates were similar regardless of cytoreductive treatment (data not shown). It may be important in future analyses to continue to report the association between ACE inhibitor and cytoreductive medication use on patient outcomes.

Of note, even among a subset of patients receiving cytoreductive treatment, the thrombotic and cardiovascular risk remained high. Approximately 1 in 4 patients treated with cytoreductive therapy experienced thrombotic events during follow-up, and nearly 1 in 3 had cardiovascular events. These data indicate an unmet clinical need in patients treated with traditional options and may in part explain why such a large proportion of patients were not receiving cytoreductive treatment during follow-up.

Limitations of this analysis are primarily related to the retrospective study design and reliance on the accuracy of the database. The PV disease diagnosis, thrombotic events, and other clinical conditions were identified using *ICD-9-CM* codes, which are subject to potential miscoding (eg, cases of secondary polycythemia may have been logged as PV). Some PV-related treatments (eg, over-the-counter aspirin, phlebotomy at blood centers) may not have been recorded in the database and could have been underrepresented in our analysis. Gaps between patient visits could be long, during which time blood cell counts and other clinical measures were unavailable; this may have precluded an ability to observe long-term changes in a consistent group of patients. Only 1 WBC count ≥11 × 10^9^/L was required for patients to have an elevated status, and it could have been caused by an acute illness. However, previous data suggest that this cutoff is important. In an analysis of the CYTO-PV study, WBC count ≥11 × 10^9^/L was associated with a 3.9-fold increased risk of major thrombosis compared with WBC count < 7 × 10^9^/L (*P* = 0.02) [25]. Furthermore, blood count analyses were incomplete because data were not available for an informative analysis of platelet counts. This VHA patient population was almost entirely male, precluding an analysis of treatment and management effects on thrombotic and cardiovascular events in female patients. Finally, the exploratory nature of the analysis precluded formal statistical analyses.

## Conclusions

This retrospective analysis of the VHA population identified a substantial burden of thrombotic and cardiovascular events among 7718 patients with PV managed between 2005 and 2012, before publication of the CYTO-PV results. Approximately 9 in 10 patients had elevated hematocrit levels and 1 in 3 had elevated WBC counts, which may increase the risk of PV-related clinical complications. The prevalence of additional cardiovascular risk factors was high, placing this population at a greater risk for thrombotic events. Surprisingly, many patients did not have documented treatment with cytoreductive therapy or phlebotomy. Collectively, these data suggest that some patients with PV in the VHA have unmet clinical needs that may be ameliorated with the use of both traditional and targeted cytoreductive treatment options. Recent clinical practice guidelines from the National Comprehensive Cancer Network provide specific treatment recommendations based on PV disease severity and response history [28].

## Additional file

Additional file 1: **Table S1.** Thrombotic and Cardiovascular Event Codes. This table presents the *ICD-9-CM* codes for thrombotic and cardiovascular events employed in the study. (PDF 61 kb)

## Abbreviations

CYTO-PV: Cytoreductive Therapy in Polycythemia Vera
ECLAP: European Collaboration on Low-Dose Aspirin in Polycythemia Vera
FDA: US Food and Drug Administration
*ICD-9-CM*: *International Classification of Diseases, Ninth Edition, Clinical Modification*
MPN: Myeloproliferative neoplasm
PV: Polycythemia vera
SD: Standard deviation
VHA: Veterans Health Administration
WBC: White blood cell
