# Supplementary material for: Cytoreductive treatment patterns among US veterans with polycythemia vera
Source: BMC Cancer. 2018 May 4;18:528. doi: 10.1186/s12885-018-4422-6 (PMC5935975; doi:10.1186/s12885-018-4422-6)
Supplement: Supplementary file 1 — Table S1. Thrombotic and Cardiovascular Event Codes. This table presents the ICD-9-CM codes for thrombotic and cardiovascular events employed in the study. (PDF 61 kb) [file 12885_2018_4422_MOESM1_ESM.pdf]

Cytoreductive Treatment Patterns Among US Veterans With Polycythemia Vera

Shreekant Parasuraman, BPharm, PhD,[1] Jingbo Yu, MHA, PhD,[1] Dilan Paranagama, PhD,[1] Sulena Shrestha, MPH,[2] Li Wang, PhD,[2] Onur Baser, PhD,[2,3] Robyn Scherber, MD, MPH[4,5]

[1]Incyte Corporation, Wilmington, DE; [2]STATinMED Research, Plano, TX; [3]Center for Innovation & Outcomes Research (CIOR), Columbia University, New York, NY; [4]Oregon Health and Sciences University, Portland, OR; [5]Department of Hematology and Oncology, Mayo Clinic, Scottsdale, AZ

Supplemental Table

Supplemental Table. Thrombotic and Cardiovascular Event Codes

Event	*ICD-9-CM* Code
Thrombotic	
Acute myocardial infarction	410.xx
Deep vein thrombosis	451.1, 453.x
Ischemic stroke	434, 436, 437.0, 437.1, 438, 997.02
Peripheral arterial thrombosis	444.0, 444.1, 444.22, 444.81, 444.89, 445.02, 445.81
Pulmonary embolism	415.1x
Superficial thrombophlebitis	451.0, 451.82
Transient ischemic attack	435
Cardiovascular	
Acute myocardial infarction	410.xx
Coronary artery bypass graft	CPT-4 codes: 33503-33545
Heart failure	425.xx, 429.2, 428.xx, 429.9
Ischemic stroke	434, 436, 437.0, 437.1, 438, 997.02
Percutaneous coronary intervention	00.66, 36.09
Transient ischemic attack	435
Unstable angina	411.1, 411.81, 411.89

ICD-9-CM, International Classification of Diseases, Ninth Edition, Clinical Modification.
